# The behavioral urgency of objects approaching your avatar

**DOI:** 10.3758/s13414-015-0966-6

**Published:** 2015-08-01

**Authors:** Daniel Schreij, Christian N. L. Olivers

**Affiliations:** Department of Cognitive Psychology, VU University, Amsterdam, The Netherlands; Department of Cognitive Psychology, Vrije Universiteit, Van der Boechorststraat 1, 1081 BT Amsterdam, The Netherlands

**Keywords:** Embodied perception, Object-based attention, Visual perspective taking

## Abstract

The behavioral-urgency hypothesis (Franconeri & Simons, *Psychological Science*, *19*, 686–692, 2003) states that dynamic visual properties capture human visual attention if they signal the need for immediate action. The seminal example is the potential collision of a looming object with one’s body. However, humans are also capable of identifying with entities outside one’s own body. Here we report evidence that behavioral urgency transfers to an avatar in a simple 2-D computer game. By controlling the avatar, the participant responded to shape changes of the target in a visual search task. Simultaneously, and completely irrelevant to the task, one of the objects on screen could move. Responses were overall fastest when the target happened to be the moving object and was on a collision course with the avatar, as compared to when the moving target just passed by the avatar or moved away from it. The effects on search efficiency were less consistent, except that search was more efficient overall whenever a target moved. Moreover, response speeding was frequently accompanied by an increase in errors, consistent with recent evidence that the urgency of looming is at least to a large extent expressed in response processes rather than in perceptual selection of the looming object. Thus, a general version of the behavioral-urgency hypothesis also holds for external entities with which the observer can identify.

Intuitively, we give perceptual priority to objects that move toward us. Indeed, Franconeri and Simons (2003) found that looming targets were detected more rapidly and more efficiently than stationary targets. Because a receding stimulus (i.e., the opposite of looming) did not show these signs of capture (but see Abrams & Christ, 2005; Skarratt, Cole, & Gellatly, 2009; Skarratt, Gellatly, Cole, Pilling, & Hulleman, 2014), Franconeri and Simons proposed the *behavioral-urgency hypothesis.* This hypothesis states that dynamic events are most likely capture attention when they signal potential behavioral urgency—that is, when they require immediate action, for example because they are possibly hazardous to the observer’s physical integrity. A rapidly looming object could cause injury, and thus calls for immediate evasive action, whereas a receding or otherwise moving object does not necessarily. Lin, Franconeri, and Enns (2008) provided further support for this theory by directly comparing a condition in which the object would loom toward the observer and one in which the object would loom but would appear to pass by the observer (see also Lewis & Neider, 2015). Both types of stimulus featured a retinal expansion, yet only the approaching stimulus captured attention, expressed by faster response times (RTs) and shallower search slopes when the moving stimulus was the target. Schmuckler, Collimore, and Dannemiller (2007) reported similar findings with 5-month-old infants.

A central assumption of the behavioral-urgency hypothesis is that priority assignment occurs automatically (as in fast and unintentional, driven by ingrained and potentially hardwired mechanisms). Direct support for this comes from the way in which attentional capture tasks are designed (Yantis, 1993; Yantis & Jonides, 1984), in which observers are asked to search a display for a target object that is defined by a particular feature (e.g., shape or category). The dynamic feature of interest, such as looming, is then varied independent of this target-defining property. It can coincide with the target, but it is as likely to coincide with any of the other items in the display. Thus, the dynamic feature becomes irrelevant to the task. If this feature still modulates performance (i.e., people respond more rapidly to the target when it is dynamic, and/or more slowly when one of the distractors is dynamic), it must have gained priority in an automatic fashion. That is, despite its irrelevance to the task, the feature is apparently still relevant to the *observer*, as is indeed the case for looming.

The automaticity of processing notwithstanding, Skarratt et al. (2014) recently raised the possibility that the processing advantage enjoyed by looming motion is mediated not so much by attentional mechanisms as by postattentional, response preparation processes. In one of their earlier studies, Skarratt et al. (2009) found that both looming and receding stimuli were equivalent in attracting attention. The slopes of the search functions for these two stimulus types were shallower than those for static stimuli, yet were equal to each other. If looming stimuli are selected more efficiently by attention than are receding stimuli, one would expect the slope for looming stimuli to be shallower than that of receding stimuli. Nevertheless, an important difference was that the overall RTs for looming stimuli were still shorter than those for receding ones. This additive pattern led Skarratt et al. (2014) to argue that although attentional capture might occur for dynamic stimuli in general, the response benefits for looming stimuli might be particularly caused by priming of the visuomotor system. Skarratt et al. (2014) found further evidence for this in a temporal-order judgment task (TOJ) using looming stimuli. The TOJ task provides a perceptual measure of prior entry (Shore, Spence, & Klein, 2001; Spence, Shore, & Klein, 2001; Weiss & Scharlau, 2011), because attended stimuli are perceived earlier in time than nonattended stimuli when both are presented simultaneously (Stelmach & Herdman, 1991). Skarratt et al. (2014) reasoned that if the looming advantage is mediated by the motor system rather than by attention, the TOJ should yield no differences between looming and receding stimuli. They indeed found this to be the case: Moving objects led to prior entry, but the amounts did not differ for the two types of motion direction (looming or receding). Together with the overall speeding of RTs in the irrelevant feature search, this suggests at least a large role for response preparation processes in speeding performance under conditions of behavioral urgency.

## The present study: Dissociating behavioral urgency from one’s own body

Here we further investigated the behavioral-urgency hypothesis. In our view, the strength of this hypothesis is not so much that it assumes a strong role of behavioral relevance in attentional capture (after all, many accounts assume that mechanisms of prioritization have evolved exactly because they turned out to be adaptive in avoiding harm or spotting useful information), but that it opens up a way of investigating *how sophisticated* our behavioral-urgency detection mechanisms are. To illustrate, a simple retinal expansion detection algorithm may be a very crude but still useful method of implementing the behavioral urgency of an approaching object (which works fine for, e.g., the normal house fly; Marr, 1982). What Lin et al. (2008) have shown is that for humans, the system apparently is more sophisticated than that, using higher-level algorithms to detect not only expanding objects, but also whether an object really requires action, or whether it will pass without consequences. Here we take this idea a step further. Humans have the unique ability to identify with other entities. In fact, we often imagine being somebody else, or even something else. For instance, when we read a book we experience events from the view of the story’s protagonist. The ability to identify with other entities probably also underlies what is known as *visual perspective taking*, in which participants are asked to imagine viewing the scene from another person’s perspective and to judge, for example, object visibility and position for that person (Amorim, 2003; Amorim, Glasauer, Corpinot, & Berthoz, 1997; Creem et al., 2001; David et al., 2006; Flavell, Everett, Croft, & Flavell, 1981; Michelon & Zacks, 2006; Salatas & Flavell, 1976).

Work by Samson, Apperly, Braithwaite, Andrews, and Scott (2010) has moreover shown that observers may automatically adopt another person’s viewpoint (even when it is irrelevant to the task). When observers were asked to judge the number of dots they saw, RTs were influenced by the number of dots that a person pictured in the display could see (however, see Santiesteban, Catmur, Hopkins, Bird, & Heyes, 2014, for evidence that this may be merely an effect of directing attention to specific dots—comparable to what is known as *gaze cueing*, Friesen & Kingstone, 1998; see also Cole, Smith, & Atkinson, 2015).

Our study did not concern visual perspective taking, as such. Visual perspective taking is just one example of how people identify with an external entity. Another case in which this ability becomes clear is when people control an object (such as the car one drives) or a character in a computer game (referred to as an *avatar*). Work by Yee and Bailenson (2007, 2009) and Pena, Hancock, and Merola (2009) has shown that participants tune their behavior to the virtual appearance of their avatar (called the *Proteus effect*). For example, if their avatar is taller than other virtual characters, participants become more confident in virtual negotiations. The present study focused on the question of whether we also adapt our attentional and/or motor priorities in relation to an external entity that we control. More specifically, in the present study we investigated whether objects that approach an observer’s avatar, rather than the observer him- or herself, capture attention. If so, this would lend further support to the idea that attentional and/or motor priorities depend on high-level cognitive representations of what is currently behaviorally urgent, rather than lower-level hardwired motion detection mechanisms such as simple retinal expansion sensors. To our knowledge, no studies have yet investigated whether attentional effects can also transfer to an avatar. Especially important for current theories would be the demonstration that even what are considered *automatic* processes of prioritization can transfer to external entities, since this would imply that although a process itself may be automatic, the representation to which it is assigned may change.

### The “Whack the Egg” task

**Fig. 1:**
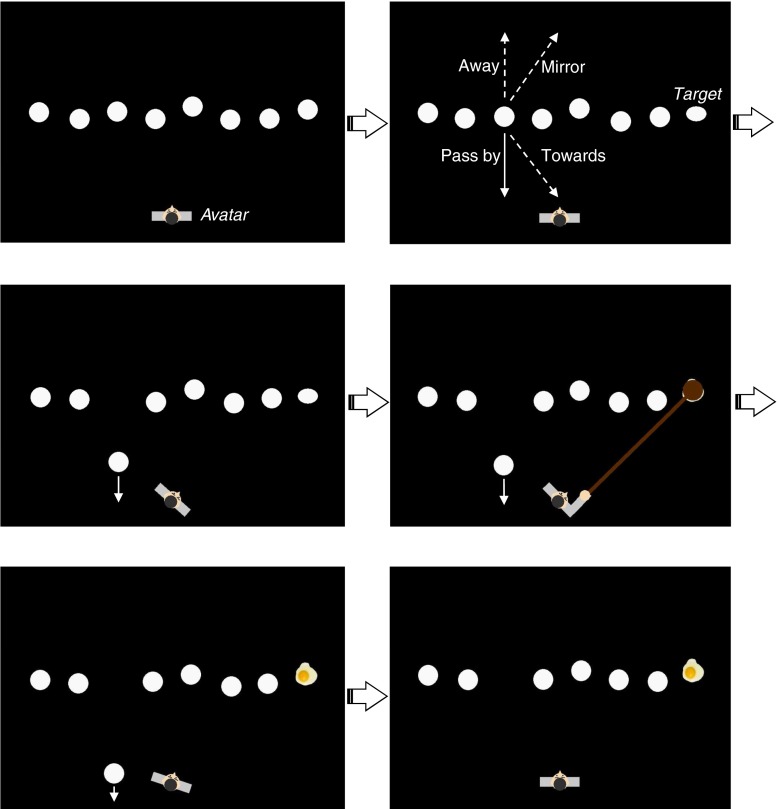
Illustration of a (pass-by) trial. The participant’s avatar was located at the bottom of the screen; the task was to rotate the avatar to align it with the egg-shaped target, after which the avatar would smash it. Simultaneous with the target appearance, one of the objects could start moving in one of the indicated directions. The moving item could be either the target or a distractor. After hitting the target, the participant had to rotate the avatar back to the middle position to initiate the next trial. Labels and arrows of course were not visible in the real trials

To investigate whether behavioral urgency applies to external but still relevant entities, we adapted Lin et al.’s (2008) visual search task to a 2-D computer game that we refer to as “Whack the Egg.” The series of events is illustrated in Fig. 1. As in Lin et al., participants searched an array of circular shapes for an egg-shaped target. However, here they responded by rapidly rotating an avatar toward the egg, as well as by indicating its orientation (upright or lying down). The avatar was a character viewed from the top. To encourage the feeling of unity, the avatar was under the continuous control of the observer throughout a trial and responded in real time to keypresses. To add to the game play, the avatar would shatter the egg with a hammer once it was correctly aligned with the target, with befitting accompanying sounds. Although a simpler experimental setup might have been possible, we deliberately chose this implementation in order to increase participant engagement with the avatar and task. At the same time, we avoided a more realistic, and therefore more complex, environment in order to retain maximal control over the experimental variables.

Concurrent with the revelation of the target, any one of the objects present in the search field could start moving, an event that did not correlate with the target (which observers were explicitly made aware of). Since the movement of the object, as well as its direction, was completely uninformative about the target’s position or orientation, any motion-related advantage should be regarded as automatic. The crucial condition was when the object moved straight toward the avatar, as compared to a number of control conditions. These control conditions included a condition in which the object moved in the general direction of the avatar, but would pass it by, as well as the mirrored versions of the toward- and pass-by directions. Here the movement followed the exact same trajectories, but always away from the avatar, in order to control for other potential (e.g., visual) differences between the two movement directions. To control for motion-related visibility issues, the remaining objects were also continuously jiggling around their respective positions.

Our predictions were as follows: If an approaching movement toward one’s external representation is prioritized, then we should observe faster responses when the object turns out to be the target. In contrast, if looming toward the observer’s own visual system is essential, no signs of prioritization should be found. In addition, prioritization might operate on perceptual selection, on response preparation, or both. In the first case, we should see an improvement in terms of search slopes for targets moving toward the avatar. In the latter case, improvements should be limited to overall RT.

**Fig. 2:**
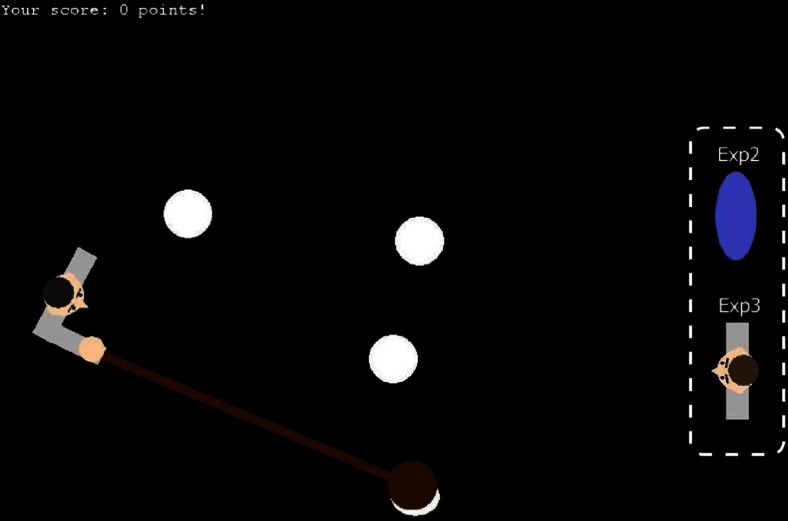
Example display for Experiments 2 and 3. As compared to Experiment 1, the whole search display was rotated 90 deg, and the avatar was now placed at the left or right side of the screen instead of only at the bottom. On the opposite side from the avatar a blue oval was positioned in Experiment 2, and an inanimate avatar image in Experiment 3

We report three experiments that showed the same overall pattern. In Experiment 1, the avatar was the only object present toward which the search items could move. To control for the idea that priority might be given to items approaching any salient stimulus at the edge of the screen, and not necessarily an avatar, Experiment 2 included a simple but salient inanimate object (a uniquely colored oval) at the opposite side of the screen, to control for any visual presence at the display side. In Experiment 3, this inanimate object was replaced with an avatar look-a-like, which shared the exact visual features with the avatar but was still inanimate. In this way, we could assess whether actually controlling the avatar is crucial, or whether looking like a human figure is in itself sufficient. We point out that the player’s own avatar only started moving at a buttonpress or mouse movement, from which moment the RT was recorded. Hence, our measurements were not affected by the avatar’s motion itself. We furthermore decided to turn the displays by 90 deg in Experiments 2 and 3 (see Fig. 2), to control for the possibility that the downward motion in the displays of Experiment 1 was interpreted as motion toward the observer’s own body (e.g., toward the legs under the table). Furthermore, the task of controlling the avatar and responding to the targets was made easier, in order to reduce the relatively large number of errors found in Experiment 1.

## Experiments

### Method

#### Participants

Fifty-four students from the VU University between 18 and 30 years old (average 24) participated, of which 18 (16 female, two male) in Experiment 1, 20 (eight female, 12 male) in Experiment 2, and 16 in Experiment 3 (ten female, six male). They signed informed consent and received course credits or money. All testing was done in accordance with the ethical guidelines of the faculty, as assessed by the local ethical review board. All participants reported normal or corrected-to-normal vision and were naïve as to the purpose of the experiment.

#### Apparatus

The experiments were run in a dimly lit cubicle, on a PC with a 19-in. CRT screen (1,024 × 768 resolution, 120 Hz), viewed from a 70-cm distance. Stimulus presentation and response recording were done in E-Prime 1.2 (Psychology Software Tools, Pittsburgh, PA, 2012).

#### Stimuli

In *Experiment*1, the search items on the display consisted of four or eight white circles [1.92 degree of visual angle (dva), CIE(.291, .350), 100 cd/m^2^] positioned on the horizontal meridian of the screen. In *Experiments*2*and*3, these circles were aligned along the vertical meridian and maximally amounted to six. All circles in the search display were slightly vibrating (randomly jiggling 2 pixels per frame along the *x*- and *y*-axes) around their position, in an attempt to (a) add to the gameplay, (b) mask the target shape change, and (c) mask the motion onset of the moving element. A target was either a horizontal or vertical oval with, respectively, half the height or the width of a distractor circle. In Experiment 1, the avatar (2.78 dva wide) was positioned at the bottom of the screen, centered on the horizontal axis. It consisted of gray shoulders, a skin-colored head, and black hair. In Experiments 2 and 3, the avatar was presented at the center of the left or right edge of the screen (alternating per block and with the initial side counterbalanced across participants) and faced the screen center. In Experiment 2, a dark blue oval [50 × 100 pixels, CIE(.16, .05), 29 cd/m^2^] was placed at the opposite edge of the screen from where the avatar was located, and in Experiment 3 this blue oval was replaced by an inactive copy of the avatar facing the center of the screen.

#### Design

Experiment 1 consisted of nine blocks containing 120 trials, of which the first block was practice. The first factor was Set Size, which could be 4 (40 trials) or 8 (80 trials). The second factor was Moving Item: There could either be no moving item (*none*, 20 % of all trials) or a moving item (80 % of trials) that was either a distractor circle (*distractor*) or target oval (*target*). The moving item was the target on one quarter of the trials when the set size was 4 (eight out of 32 trials), and on one eighth of the trials when set size was 8 (eight out of 64 trials). One of the items was randomly selected to be the target. The third factor was Movement Direction. An item could move in one of four possible directions, with each direction occurring equally often (eight trials for each direction when the set size was 4, and 16 trials when the set size was 8). On *toward-avatar* trials, the item would move directly toward the avatar. On *pass-by-avatar* trials, the item moved toward the side of the avatar, but in a direction that would miss it. On *toward-opposite* and *pass-by-opposite* trials, the item moved in the same manner as on toward and pass-by trials, but now always toward the opposite side of the screen. All of the above factors were mixed within blocks.

Experiments 2 and 3 consisted of 11 blocks containing 100 trials, of which the first block was practice. The factor Set Size could be 4 (40 trials) or 6 (60 trials). The maximum set size was reduced because the vertical meridian was less long than the horizontal, and thus allowed fewer items to be placed with sufficient space between them. The moving item was the target on one quarter of the trials when the set size was 4 (eight out of 32 trials), and one sixth of the trials when the set size was 6 (eight out of 48 trials). In all experiments, all of the above factors were mixed within blocks.

#### Procedure

Participants were given verbal and written instructions about the task before they commenced the experiment. They were made aware that, when present, the moving element was unlikely to be a target, although on certain occasions this was possible. No reference was made to identifying with the avatar. We only explained to participants what the task was and how to operate the avatar.

A trial started with the presentation of the avatar, which was facing the center of the screen. After 500 ms, the array of vibrating circles appeared. After approximately 2 s, one of the circles turned into the egg shape and, simultaneously, it or any other circle could start moving from its position, with a speed of 10 dva per second. At the same time, response measurements started. Whenever a moving circle or oval reached the edge of the screen or was about to collide with the avatar, it faded into the background. Thus, the item never actually collided with the avatar, and there were no negative consequences or penalties in the game when it reached the avatar.

In Experiment 1, the avatar had to be rotated to the oval target by pressing the “1,” “7,” “3,” or “9” key on the numeric keypad, depending on the target’s location and orientation. When the target was located to the left of the avatar, the avatar had to be rotated with the “7” key when this target was a vertical oval, or with “1” when it was horizontal. Likewise, the avatar had to be moved with the “9” or “3” key when the target was located to its right. The RT was defined as the time from target revelation to a participant’s first keypress. Any subsequent keypresses were not important for the data analysis. Since their control over the avatar was continuous, participants had to hold down the correct key until the nose of the avatar was aligned with the target, after which the avatar would automatically smash the egg with an emerging hammer.

In Experiment 1, choosing the correct response key out of the four possible keys proved to be quite difficult for participants, resulting in high error rates. To simplify the task, we let participants respond with the mouse instead of the keyboard in Experiments 2 and 3, and used two separate “modalities” of the mouse to respond to the orientation and position of the target. Participants could rotate the avatar by sliding the mouse up or down, and they pressed the left mouse button if the oval was horizontal and the right button if it was vertical. The RT was measured as the time between the revelation of the target oval and the buttonpress. The avatar would not strike until it faced the target and one of the mouse buttons had been pressed. These two actions could be done simultaneously and did not need to be performed in a specific order. A response was counted as incorrect if the wrong mouse button was pressed or when the avatar was initially rotated in the opposite direction from the one in which the target oval was located.

A high- or low-frequency sound was played for a correct or incorrect response, after which an image of a fried egg would appear at the previous location of the egg shape, following a correct response, and an image of a dizzy duckling, after an incorrect response. Participants then had to rotate the avatar back to its starting orientation to start the next trial. When a participant initially rotated the avatar in the wrong direction (e.g., to the opposite half of the screen from where the target oval was located) or pressed a button that did not correspond to the orientation of the oval, the response was registered as being incorrect. To promote the gameplay, participants still had to finish the trial in this case, by aligning the avatar with the egg to smash it and then returning to the starting orientation. In addition, to motivate participants to respond as quickly as possible, the positive or negative feedback depended on a moving RT criterion of 200 ms above the running average of the past ten trials (starting with 1,500 ms at each block). When participants responded correctly, but too slow, the response feedback was given as incorrect (in the form of the oval turning to a small chick and a short quacking sound), but for purposes of the analyses, the response was registered as correct. There was a break after each block, and the experiments took about 70 to 90 min.

## Results

**Fig. 3:**
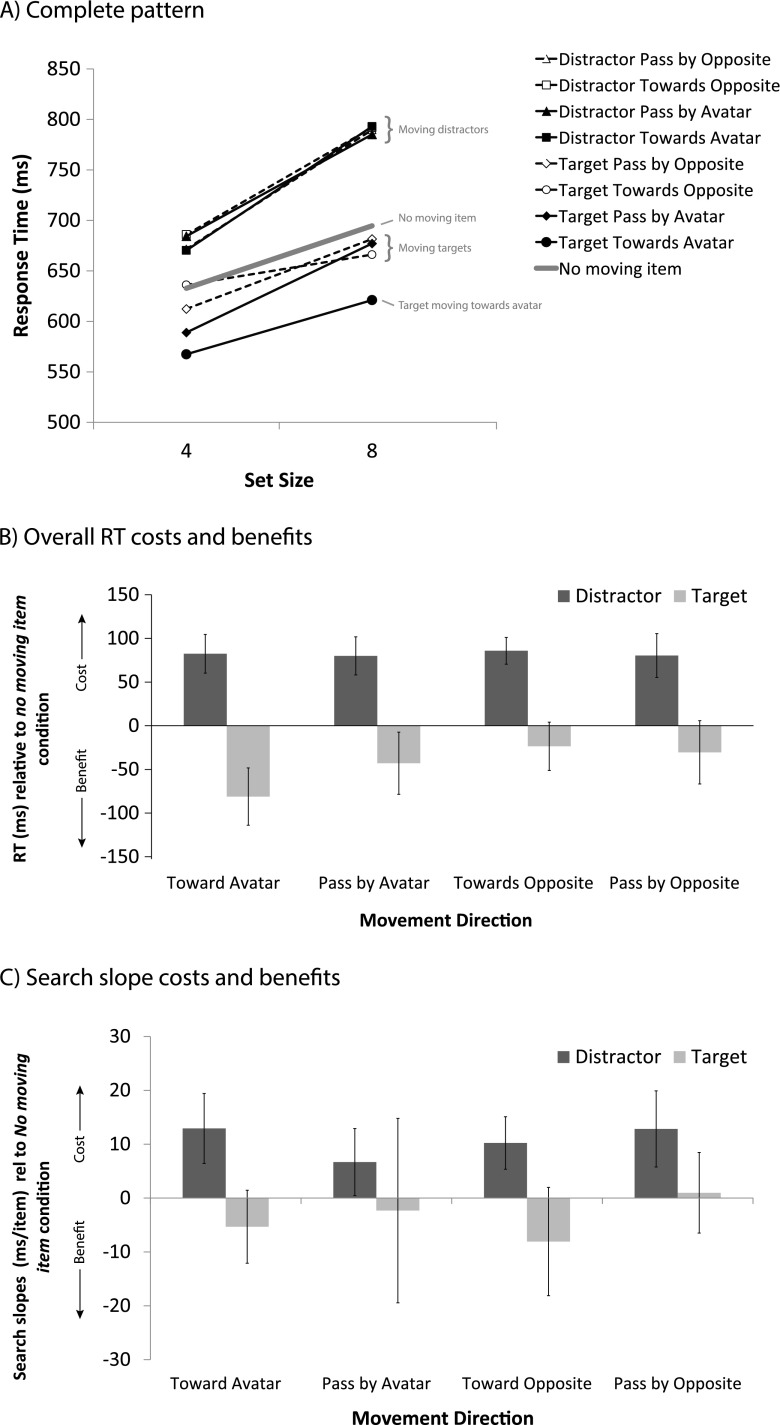
Results of Experiment 1. (**A**) Mean response times (RTs) for conditions in which the moving item was absent or was a distractor or the target, plotted against set size. (**B**) RT costs (for moving distractors, positive) and benefits (moving targets, negative) for each movement direction, relative to the condition in which no moving item was present. (**C**) Search slopes for these same items relative to the slopes of the condition in which no moving item was present. Error bars represent the within-subjects 95 % confidence intervals of the mean differences relative to the no-movement baseline

**Fig. 4:**
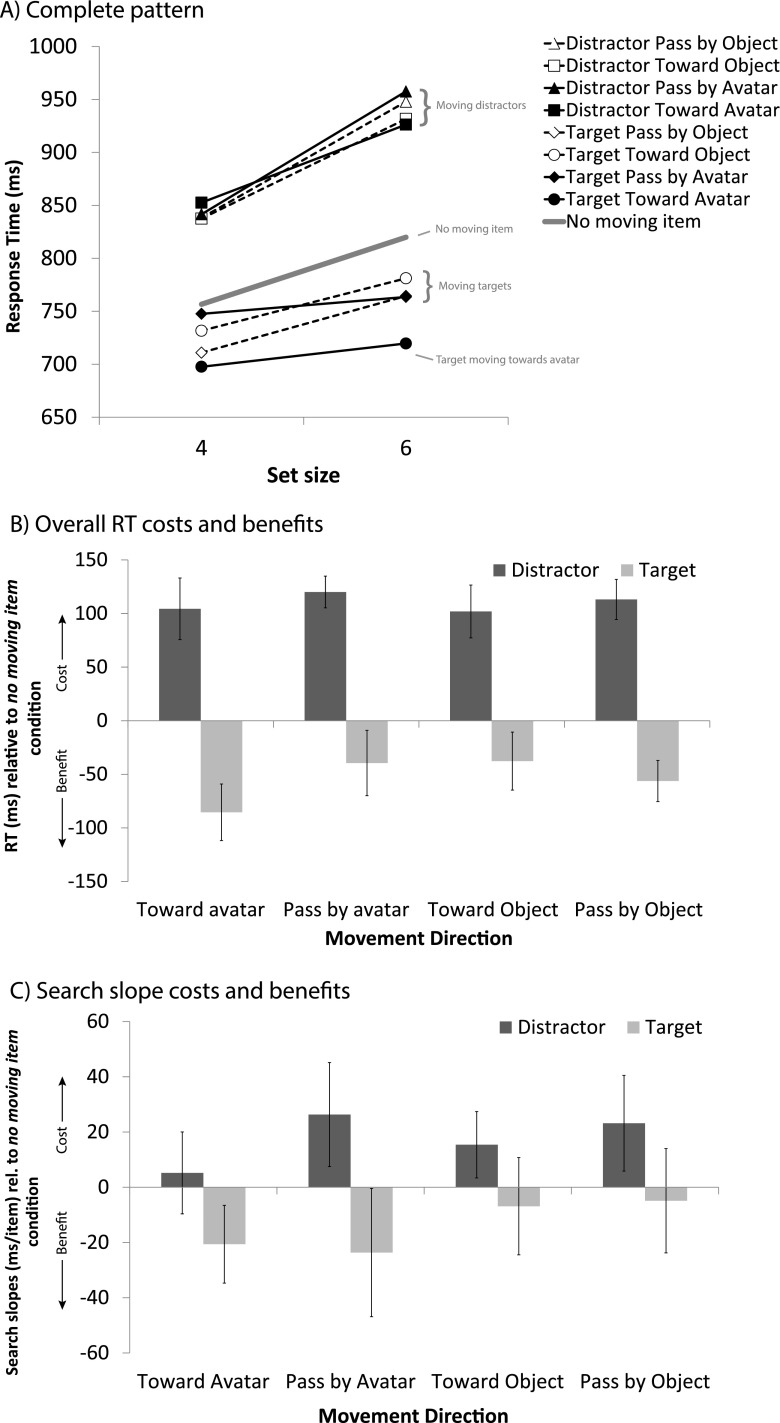
Results of Experiment 2. (**A**) Mean response times (RTs) for conditions in which the moving item was absent or was a distractor or the target, plotted against set size. (**B**) RT costs (for moving distractors, positive) and benefits (moving targets, negative) for each movement direction, relative to the condition in which no moving item was present. (**C**) Search slopes for these same items relative to the slopes of the condition in which no moving item was present. Error bars represent the within-subjects 95 % confidence intervals of the mean differences relative to the no-movement baseline

**Fig. 5:**
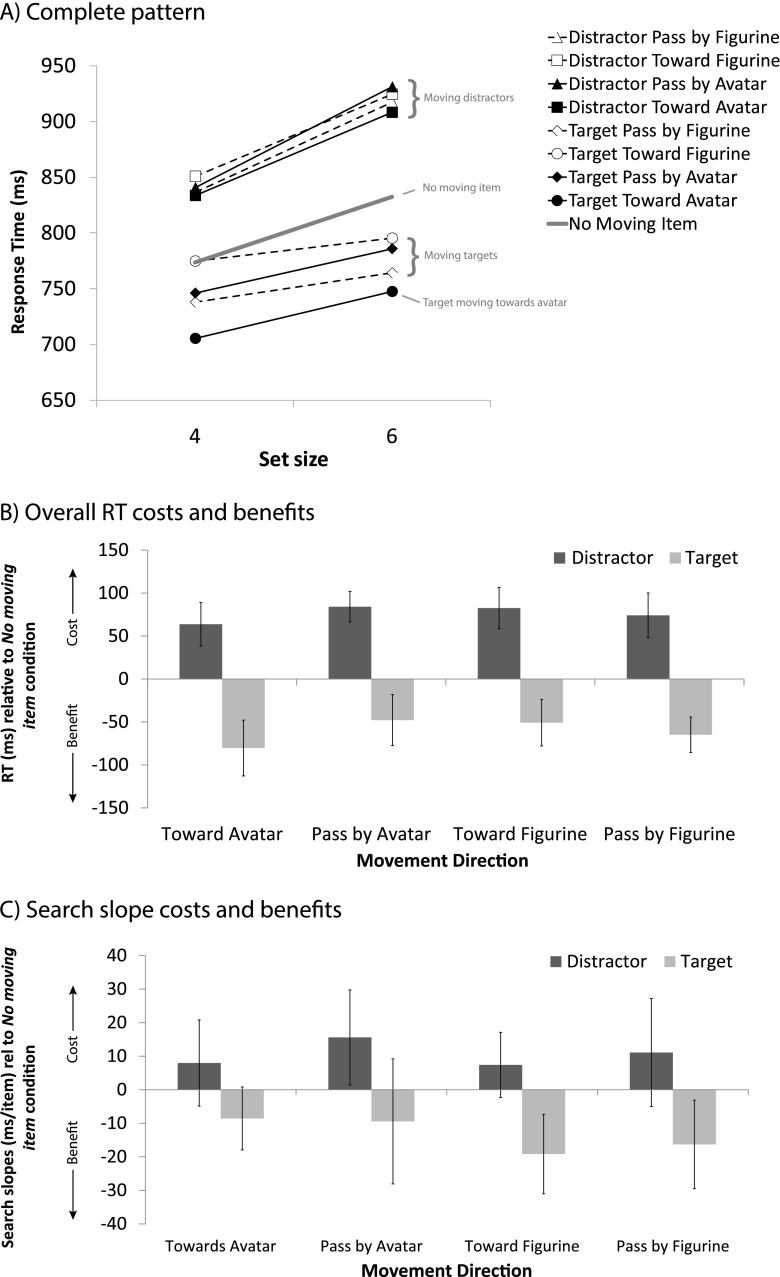
Results of Experiment 3. (**A**) Mean response times (RTs) for conditions in which the moving item was absent or was a distractor or the target, plotted against set size. (**B**) RT costs (for moving distractors, positive) and benefits (moving targets, negative) for each movement direction, relative to the condition in which no moving item was present. (**C**) Search slopes for these same items relative to the slopes of the condition in which no moving item was present. Error bars represent the within-subjects 95 % confidence intervals of the mean differences relative to the no-movement baseline

**Fig. 6:**
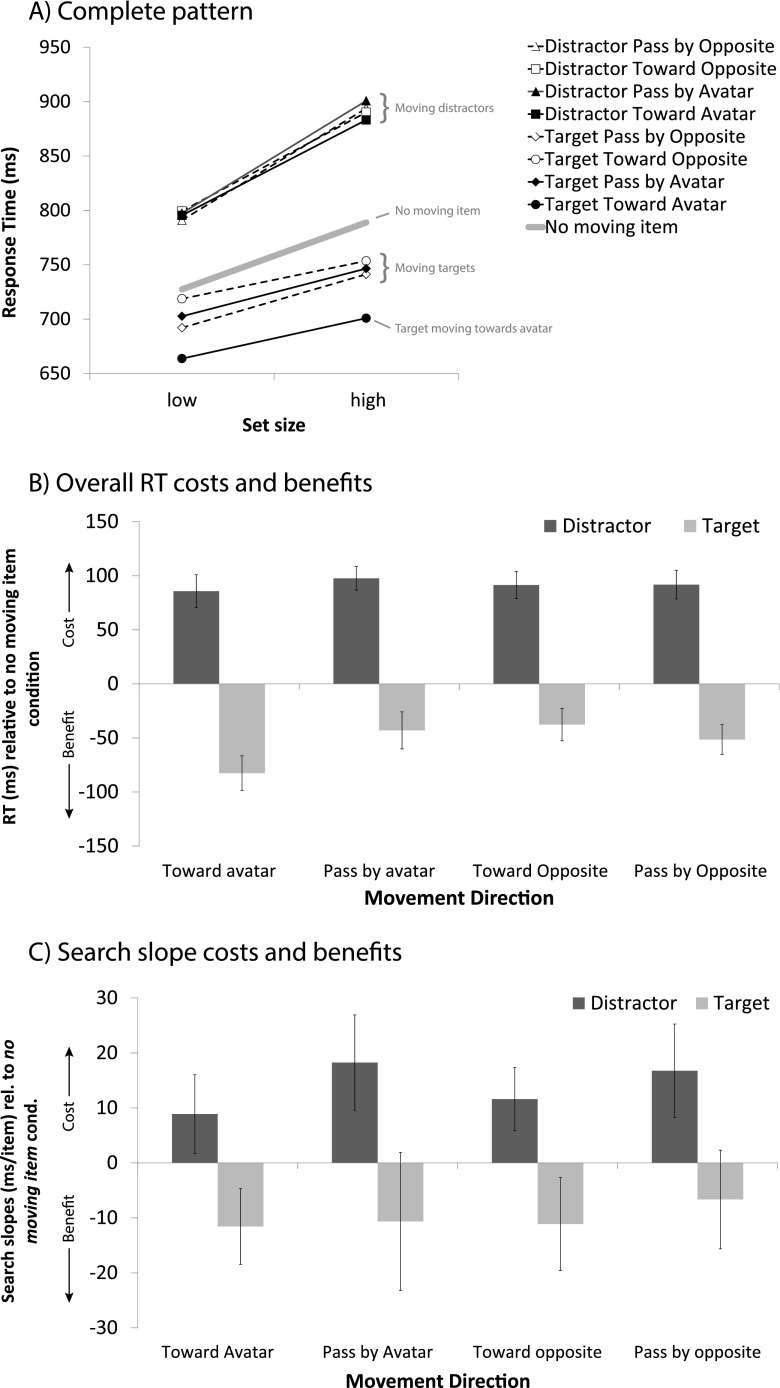
Results collapsed over Experiments 1–3. (**A**) Mean response times (RTs) for conditions in which the moving item was absent or was a distractor or the target, plotted against low (4) and high (8 in Exp. 1, 6 in Exps. 2 and 3) set size. (**B**) RT costs (for moving distractors, positive) and benefits (moving targets, negative) for each movement direction, relative to the condition in which no moving item was present. (**C**) Search slopes for these same items relative to the slopes of the condition in which no moving item was present. Error bars represent the within-subjects 95 % confidence intervals of the mean differences relative to the no-movement baseline

The measure of interest was the target identification RT (corresponding to the buttonpress). The mouse movement latency did not provide a clean measure, since the necessity and extent of moving the mouse differed per condition. In Experiment 1, four participants who made more than 30 % errors were removed from the data set. Trials with erroneous responses (19 % in Exp. 1, 5.5 % in Exp. 2, and 9.6 % in Exp. 3) were removed from the data set, and so were trials with responses that differed more than 2.5 *SD*s from the participant’s mean (2.5 % in Exp. 1, 2.6 % in Exp. 2, 2.5 % in Exp. 3). The resulting RTs are displayed in Figs. 3a, 4a, and 5a. For convenience, and because the results are very similar for all experiments, Fig. 6a displays the results collapsed across experiments.

To assess the effects of movement direction on search, we first performed analyses of variance (ANOVAs) for each experiment with Set Size (low, high), Moving Item (distractor, target), and Movement Direction (toward avatar, pass by avatar, toward opposite, pass by opposite) as factors. Note that the no-movement condition was left out of this particular analysis, since it could not be fully crossed with the other factors (as it had no movement direction). This analysis revealed significant main effects in all experiments of set size [Exp. 1, *F*(1, 13) = 179.81, *p* < .005, *η*_p_^2^ = .93; Exp. 2, *F*(1, 19) = 159.88, *p* < .001, *η*_p_^2^ = .89; Exp. 3, *F*(1, 15) = 158.80, *p* < .001, *η*_p_^2^ = .91], moving item [Exp. 1, *F*(1, 13) = 59.47, *p* < .005, *η*_p_^2^ = .82; Exp. 2, *F*(1, 19) = 204.53, *p* < .001, *η*_p_^2^ = .92; Exp. 3, *F*(1, 15) = 116.92, *p* < .001, *η*_p_^2^ = .87], and movement direction [Exp. 1, *F*(3, 39) = 6.41, *p* < .005, *η*_p_^2^ = .33; Exp. 2, *F*(3, 57) = 4.99, *p* < .005, *η*_p_^2^ = .21; Exp. 3, *F*(3, 45) = 4.34, *p* < .05, *η*_p_^2^ = .23]. Responses were overall faster for the lower set size, were faster when the moving item was the target, and were fastest when the moving item moved toward the avatar. All experiments also showed a significant Moving Item × Set Size interaction [Exp. 1, *F*(1, 13) = 16.76, *p* < .005, *η*_p_^2^ = .56; Exp. 2, *F*(1, 19) = 46.126, *p* < .005, *η*_p_^2^ = .70; Exp. 3, *F*(1, 15) = 28.40, *p* < .005, *η*_p_^2^ = .65], reflecting the fact that the set size effects (and thus the search slopes) were smaller when the target was moving than when a distractor was moving. Furthermore, in Experiments 1 and 2 we observed a reliable Movement Direction × Moving Item interaction [Exp. 1, *F*(3, 39) = 3.09, *p* < .05, *η*_p_^2^ = .19; Exp. 2, *F*(3, 57) = 4.46, *p* < .05, *η*_p_^2^ = .19]: RTs were especially affected when the moving object was a target and moved toward the avatar. This Movement Direction × Moving Item interaction was not reliable in Experiment 3 [*F*(3, 45) = 1.20, *p* = .32, *η*_p_^2^ = .07]. The specific effects underlying these overall ANOVA results are fleshed out next.

### Relative RT effects

The RT benefits for targets moving toward the avatar become especially apparent when comparing the costs and benefits for each movement direction against the no-movement baseline condition. These are shown in Figs. 3b, 4b, and 5b for Experiments 1–3, respectively. Planned pairwise comparisons revealed that in all experiments a moving distractor caused significant RT costs in all movement directions, as compared to the no-movement condition (all *t*s > 5.24, all *p*s < .005). When the target was the moving object, response benefits were greatest when it was approaching the avatar [toward avatar vs. no moving item: Exp. 1, *t*(13) = 5.35, *p* < .005; Exp. 2, *t*(19) = 7.09, *p* < .005; Exp. 3, *t*(1, 15) = 3.07, *p* < .01]. There were also reliable benefits when the target passed by the avatar (pass by avatar vs. no moving item: all *t*s > 2.60, *p*s < .05), but importantly, in all cases these benefits were significantly smaller than when the target approached the avatar (toward vs. pass by avatar: *t*s > 2.28, *p*s < .05). Targets moving in the directions opposite the avatar did not elicit reliable response benefits (vs. no moving item) in Experiment 1 (*t*s < 1.84, *p*s > .09), but did so in Experiments 2 and 3 (all *t*s > 2.20, *p*s < .05). Most importantly, these benefits were all also significantly smaller than those yielded by a target that approached the avatar (all *t*s > 2.28, *p*s < .05). In Experiment 3, the difference in the RT benefits of a target moving toward the avatar versus passing by the opponent failed to reach significance by a small margin (*t*s < 1.82, *p* > .08).

The pattern of results becomes especially clear when the data are collapsed across experiments, as they are depicted in Fig. 6b. Separate ANOVAs with Experiment as a between-subjects factor revealed no interaction between Experiment and Movement Direction for either moving-item type (*F*s < 1.17, *p*s > .33), which underlines the similarity of the results between experiments. With moving targets, the main effect of movement direction [*F*(3, 138) = 17.56, *p* < .001] was significant. Separate analyses showed that all moving-target condition RTs were significantly faster than the no-movement baseline (*t*s > 5.0, *p*s < .001), but more importantly, the RTs for a target moving toward the avatar were significantly faster than those for targets moving in any of the other directions (all *t*s > 5.73, *p*s < .001).

### Slope effects

To assess search efficiency, we looked at the slope effects for all moving conditions as compared to the no-movement baseline condition, in separate 2 (movement vs. no movement) × 2 (set size low and high) ANOVAs for moving targets and moving distractors. These differences in slopes are shown in Figs. 3c, 4c, and 5c, whereas the underlying absolute slope values are listed in Table 1. The analysis revealed that in Experiments 1 and 2, in the moving-distractor condition, search was less efficient than in the no-movement baseline for each movement direction (Exp. 1, *F*s > 5.28, *p*s < .01; Exp. 2, *F*s > 7.18, *p*s < .05), except for distractors moving toward the avatar in Experiment 2 [*F*(1, 13) = 0.54, *p* = .47]. In Experiment 3, there was little effect of a moving distractor on search efficiency relative to the no-movement baseline, except for distractors passing by the avatar, in which case search was significantly less efficient [*F*(1, 15) = 5.53, *p* < .05].

**Table 1:** Slope effects (assumed to be the inspection time, in milliseconds, per item present in the display) across experiments for the various moving-object conditions

Condition	Experiment 1	Experiment 2	Experiment 3
No moving item	15	32	29
Target toward	13	11	21
Target pass by	22	8	20
Target mirror	7	25	10
Target away	17	27	13
Distractor toward	31	37	37
Distractor pass by	25	58	45
Distractor mirror	26	47	37
Distractor away	30	55	41

For search in the moving-target conditions, the results were ambiguous, with no clear pattern favoring targets specifically moving toward the avatar. In Experiment 1, we observed trends toward more efficient search for both of the toward conditions (*F*s > 2.84, *p*s < .11), regardless of whether this was toward the avatar or toward the mirror position (away from the avatar). There was little evidence for a slope benefit in the pass-by conditions (*F*s < 0.09, *p*s > .77). In Experiment 2, search in the moving-target conditions was as efficient as in the no-movement condition when the target moved away from the avatar in any direction (*F*s < 0.23, *p*s > .42), but was more efficient when the target moved in the general direction of the avatar and also when it passed by (*F*s > 4.5, *p* < .05). In Experiment 3, search for moving targets was in general more efficient than for no moving targets, especially when the target moved toward or passed the opponent [*F*(1, 15) = 7.87, *p* < .05, and *F*(1, 15) = 4.74, *p* < .05]. Figure 6c depicts the slope effect data collapsed across experiments. ANOVAs for moving distractors and targets with Movement Direction as a within-subjects and Experiment as a between-subjects factor revealed no significant effects. A separate analysis against the no-movement baseline revealed that search was overall less efficient when there was any moving distractor (*t*s > 3.15, *p*s < .001), and was just as efficient for targets moving past the avatar [*t*(48) = 1.12, *p* = .27] or in the opposite direction [*t*(48) = 1.29, *p* = .20]. Targets that either moved toward the avatar or to the equivalent location at the opposite end were found more efficiently [*t*(48) = 3.23, *p* < .005, and *t*(48) = 2.82, *p* < .01, respectively]. Taken together, the evidence thus suggests an efficiency benefit for specific motion trajectories, rather than due to the avatar. Note that targets moving either toward the avatar or in the mirrored direction on average moved more toward the center, which may have yielded a slight benefit in search efficiency.

### Errors

The error rates for all conditions in Experiments 1–3 are displayed in Table 2. ANOVAs were performed on the error rates, again with Set Size, Moving Item, and Movement Direction as factors. These revealed no significant effects for Experiment 2, but for Experiment 1 they showed that participants made significantly more errors when the moving item was a target than when it was a distractor moving item [*F*(1, 13) = 5.51, *p* < .05, *η*_p_^2^ = .30]. Errors were also significantly higher for set size 8 than for set size 4 [set size: *F*(1, 13) = 7.97, *p* < .05, *η*_p_^2^ = .38]. Additionally, errors were higher when items followed a *pass-by* or *toward-avatar* vector, as compared to their mirrored directions [movement direction: *F*(3, 39) = 5.78, *p* < .005, *η*_p_^2^ = .31]. Set size had a greater effect on errors for moving targets than for moving distractors [Moving Item × Set Size: *F*(1, 13) = 4.68, *p* = .05, *η*_p_^2^ = .27]. Furthermore, error rates increased more for targets moving in the *pass-by* and *toward-avatar* directions than for distractors moving in those same directions [Moving Item × Movement Direction: *F*(3, 39) = 3.01, *p* < .05, *η*_p_^2^ = .19]. Planned comparisons revealed that error rates were significantly higher for all conditions in which a moving item was present, as compared to when no moving item was present (*t*s > 2.45, *p*s < .03). For Experiment 3, a main effect was found for moving item [*F*(1, 15) = 8.57, *p* < .05, *η*_p_^2^ = .36]: Participants made more errors when the moving item was a target. We also observed a main effect of movement direction [*F*(3, 45) = 5.45, *p* < .05, *η*_p_^2^ = .27]. Separate comparisons revealed that participants made the most erroneous responses when an item moved toward the avatar rather than in any of the other directions [vs. pass by avatar, *t*(15) = 2.42, *p* < .05; vs. pass by opponent, *t*(15) = 2.56, *p* < .05; vs. toward opponent, *t*(15) = 2.11, *p* = .052].

**Table 2:** Error rates of Experiments 1, 2, and 3 for conditions in which the moving item was a target or a distractor, or in which no moving item was present, in combination with the possible movement directions and set sizes

Experiment 1	Movement Direction and Set Size									
	Pass by Mir.		Toward Mir.		Pass by Av.		Toward Av.		None	
Moving Item	*4*	*8*	*4*	*8*	*4*	*8*	*4*	*8*	*4*	*8*
Distractor	13 %	14 %	12 %	13 %	16 %	17 %	19 %	18 %	–	–
Target	15 %	17 %	15 %	23 %	21 %	32 %	18 %	23 %	–	–
No moving item	–	–	–	–	–	–	–	–	8 %	9 %
Experiment 2	Movement Direction and Set Size									
	Pass by Obj.		Toward Obj.		Pass by Av.		Toward Av.		None	
	*4*	*6*	*4*	*6*	*4*	*6*	*4*	*6*	*4*	*6*
Distractor	4 %	6 %	5 %	5 %	5 %	6 %	7 %	6 %	–	–
Target	7 %	5 %	7 %	5 %	6 %	7 %	9 %	7 %	–	–
No moving item	–	–	–	–	–	–	–	–	4 %	5 %
Experiment 3	Movement Direction and Set Size									
	Pass by Op.		Toward Op.		Pass by Av.		Toward Av.		None	
	*4*	*6*	*4*	*6*	*4*	*6*	*4*	*6*	*4*	*6*
Distractor	6 %	6 %	6 %	7 %	7 %	6 %	8 %	8 %	–	–
Target	8 %	10 %	8 %	7 %	11 %	11 %	12 %	14 %	–	–
No moving item	–	–	–	–	–	–	–	–	6 %	6 %

*Mir.* empty location on opposite screen side of avatar, *Av.* avatar, *Obj.* object on other side of screen, *Op.* opponent figure on other side of screen

## General discussion

We believe the data support a number of conclusions. First, moving items appear to capture attention in general, regardless of their trajectory. Moving targets led to faster responses and to overall more efficient search than conditions with moving distractors, despite the fact that the motion was irrelevant to the task. This replicates work by Franconeri and Simons (2003), who also identified the onset of two-dimensional transposition to be an attention-capturing feature. Second, the important novel finding was the additional speeding of responses for targets moving toward the observer’s avatar. Crucially, RTs were faster than for the condition in which the object passed by the avatar, or for the mirrored directions. Moreover, there was no consistent speeding for targets moving toward a salient other object in the display (the blue oval of Exp. 2, or an inanimate opponent that was identical in appearance to the avatar in Exp. 3). The findings thus show that an object is not only prioritized when it is looming toward the observer’s own body or head, but also when it is approaching an external representation of one’s self.

At the same time, we also observed an important difference with some of the earlier findings with looming stimuli: Although an approaching target led to overall RT benefits, it did not lead to consistent benefits in search efficiency over other target directions, as would be expressed in shallower search slopes. Slopes were substantially shallower for moving-target than for moving-distractor conditions, suggesting attentional capture, but this was the case for all movement directions. This lack of a clear slope effect is not unique. Looking at several previous studies investigating attentional capture, search slope benefits seemed to occur mainly for set sizes up to 4, whereas for higher set sizes the benefits were often expressed in terms of overall RTs (Franconeri & Simons, 2003; Gellatly, Cole, & Blurton, 1999; Humphreys, Olivers, & Yoon, 2006; Jonides & Yantis, 1988; Yantis & Johnson, 1990; Yantis & Jonides, 1996). In the present study we used set sizes up to 8, which is outside the range for which slope effects have typically been found. If efficiency benefits are indeed confined to smaller set sizes, this would suggest that the automatic perceptual prioritization of looming within a set of items might be contingent on observers already attending to that set. A number of findings suggest that spatial attention may be limited to four individual items at a time (see, e.g., Cowan, 2001; Olivers, 2008, for reviews). If some initial spatial attention is needed in order to detect looming (or, potentially, other properties), this would explain the limited slope effects. However, we point out that the study that directly inspired ours, by Lin et al. (2008), did find clear slope benefits for approaching targets, even though they used set sizes 3 and 6. An “attention-limited attentional capture” explanation is thus not very satisfactory, not least because it would oppose its own purpose of attentional capture.

Instead, our pattern of results connects better with the idea put forward by Skarratt and colleagues (Skarratt et al., 2009; Skarratt et al., 2014), that approaching stimuli prime the *visuomotor response system* rather than cause attentional capture. In all three experiments, the absolute RT effects for targets collision-bound with the avatar were significantly greater than in the other movement directions, whereas there was little to no difference between the slopes. Instead, slopes were overall shallower across the moving-target conditions (and steeper when distractors were moving). This suggests a two-tier mechanism—namely, one in which attention is initially captured by the motion dynamics, which is then followed by a later process in which the response is speeded when the selected stimulus also appears to be approaching.

One might argue that approaching objects did not result in shallower slopes because they never actually collided with the avatar. This might be true, but of course it is also exactly the point: If attentional capture depends on such contextual knowledge, it is no longer automatic. One might also argue that the lack of an effect on search slopes was due to a simple floor effect. However, with average slopes values around 16 ms/item for targets in the motion conditions, there was room for further improvement. Furthermore, at the other end of the RT spectrum, we observed no sign that looming distractors led to slower or less efficient search. Distractors moving toward the avatar did not interfere more with search than did a distractor moving in any of the other directions, which is not predicted from an attentional-capture stance. The lack of a distractor effect is consistent with the findings of Lin et al. (2008), who also observed equal performance for all of their moving-distractor conditions. Thus, both when the object moved toward the observer’s body and when it moved toward the observer’s avatar, prioritization of an object on a collision course was only apparent when this object was the target. One possibility is that approaching distractors also initially engage more attention (which should initially lead to additional RT costs), but this is then compensated for by speeding up the search for (and responding to) the actual target, because the observer feels the urge to react quickly.

One might expect that when participants experience an increased sense of urgency to respond, they will also tend to make more mistakes. Experiment 1 indeed showed signs of such a speed–accuracy trade-off, in that errors increased when targets were moving in the avatar’s direction in general (toward and passing by). A similar trend was present in Experiment 3, regardless of whether the item was a target or a distractor. Experiment 2 showed no significant differences in error rates. Given these inconsistencies, we are reluctant to draw too strong conclusions, but in any case, such trade-offs would provide further support for a large response-based component (i.e., an urge to act rapidly, regardless of whether it is the correct action) to behavioral-urgency effects.

One might question whether it is necessary for participants to control the avatar or whether paying attention to its location would be sufficient to elicit the prioritization effects that we found. Even though it is possible that the effect is driven by the fact that participants attend to the avatar’s location during the task, this would not necessarily be helpful. For one thing, allocating attentional resources to the avatar would diminish the resources available to find the oval target, and therefore would not be a wise decision. Furthermore, performing the task correctly did not really require maintaining updated information about the avatar. One needed to know the avatar’s initial position to determine whether and in which direction the avatar needed to be rotated (depending on where the target appeared), but this was the same for trial after trial. That said, it may well be possible that part of the identification process involves attention, in that an entity that one regards as oneself automatically attracts attention. Evidence for this has come from Sui, He, and Humphreys (2012), who found that simple geometric shapes that were labeled “you” (referring to the participant) became more salient in a subsequent visual attention task. The present effects may then reflect the fact that the moving item approached the current focus of attention, rather than that it approached a representation of the self as such. However, if a representation of the self always correlates with attending to oneself, these mechanisms would be difficult to disentangle. In fact, attention might be the very mechanism by which we “tag” representations of ourselves in outer space.

At a general level, the present findings resemble the effects of an avatar on other behavior in either a virtual or the real world. Previous studies have already shown that we are able to identify ourselves or empathize with our avatar and to adapt our behavior accordingly (Yee & Bailenson, 2007, 2009; Yee, Bailenson, & Ducheneaut, 2009). Studies into visual perspective taking have furthermore shown that we can imagine viewing a scene through the eyes of another agent and can interact with the scene as such (Amorim, 2003; Amorim et al., 1997; Creem et al., 2001; David et al., 2006; Michelon & Zacks, 2006; Zacks, Vettel, & Michelon, 2003). Here we add the finding that we adapt our visuomotor behavior to our external representative and react to stimuli that interact with this avatar as if they were interacting with our own body, despite the fact that these stimuli were completely irrelevant to the task. Furthermore, the level at which this interaction occurs appears to involve an avatar-centered spatial reference frame, since it distinguishes between approaching and passing items—something that has been referred to as “Level 2” perspective taking in the visual perspective-taking literature (with “Level 1” referring to the mere judgment whether or not an object is at all visible to the other person; Flavell et al., 1981; Salatas & Flavell, 1976). Thus, once assigned, the process operates rather automatically, but how it is assigned is subject to higher-order identification processes.

However, we emphasize that we do not claim that observers are “seeing what the avatar sees.” We do not assume that observers actually take the avatar’s visual perspective. Thus, our results do not depend on whether observers believe that the avatar can (within its virtual world) actually *see* the object or not (cf. Cole et al., 2015). Instead, we see the evidence as being consistent with the more general idea that observers identify with the avatar because it is the object that one controls. The avatar becomes, to some extent, part of oneself, an external reference, like a car can become part of the driver or a violin can become part of the musician. Anything approaching the avatar is thus like anything approaching oneself, resulting in automatic speeding of responses. In this sense, our results may also be linked to work demonstrating that objects presented near the observer’s hands are being prioritized for processing (Abrams, Davoli, Du, Knapp, & Paull, 2008; Brockmole, Davoli, Abrams, & Witt, 2013; di Pellegrino & Frassinetti, 2000; Reed, Betz, Garza, & Roberts, 2010; Reed, Grubb, & Steele, 2006) and work showing that such prioritization extends or shifts to areas that are surrounding, or are reachable by, a tool held by the observer (Berti & Frassinetti, 2000; Cardellicchio, Sinigaglia, & Costantini, 2013; Farnè, Iriki, & Ladavas, 2005; Maravita, Husain, Clarke, & Driver, 2001). Single-cell recordings on monkeys have shown, moreover, that visuotactile neurons extend their receptive fields from areas near the hand to areas reached by a tool that is controlled by the hand (Iriki, Tanaka, & Iwamura, 1996). Such findings suggest that the system prioritizes objects that are available for (i.e., invite or afford) action, and that this is the case not only for one’s own body, but also for objects controlled by the body. Receptive-field extensions may also provide a mechanism for identifying with an avatar, since an avatar could be regarded as a more extreme case of an object controlled by the hands, although it is more remote than a typical tool. Also consistent with this, remote interactions with objects have been shown to affect spatial perceptions of those objects (Davoli, Brockmole, & Witt, 2012; Lee, Lee, Carello, & Turvey, 2012).

In conclusion, humans are known to have a unique capacity to personalize ourselves with other relevant objects or people and to react to stimuli interacting with these external entities as if those stimuli were interacting with ourselves. The present findings lend support to the general behavioral-urgency hypothesis proposed by Franconeri and Simons (2003) and, more importantly, demonstrate that this hypothesis not only holds for our own body representation, but can also be extended to external entities controlled by us. This reinforces the notion put forward by the behavioral-urgency hypothesis that more likely it is the perceived necessity for immediate action that makes us respond faster, rather than pure physical stimulus properties such as looming or expanding.
